# Nutraceutical Approach to Chronic Osteoarthritis: From Molecular Research to Clinical Evidence

**DOI:** 10.3390/ijms222312920

**Published:** 2021-11-29

**Authors:** Alessandro Colletti, Arrigo F. G. Cicero

**Affiliations:** 1 Department of Science and Drug Technology, University of Turin, 10125 Turin, Italy; alessandro.colletti@unito.it; 2 Italian Nutraceutical Society (SINut), 40138 Bologna, Italy; 3 IRCCS AOU Policlinico S. Orsola-Malpighi, Medical and Surgical Sciences Department, University of Bologna, 40138 Bologna, Italy

**Keywords:** osteoarthritis, nutraceuticals, glucosamine, chondroitin, collagen, methylsulfonylmethane, vitamin C, vitamin D, hyaluronic acid

## Abstract

Osteoarthritis (OA) is a degenerative inflammatory condition of the joint cartilage that currently affects approximately 58 million adults in the world. It is characterized by pain, stiffness, and a reduced range of motion with regard to the arthritic joints. These symptoms can cause in the long term a greater risk of overweight/obesity, diabetes mellitus, and falls and fractures. Although the current guidelines for the treatment of OA suggest, as the gold standard for this condition, pharmacological treatment characterized by non-steroidal anti-inflammatory drugs (NSAID), opioids, and cyclooxygenase (COX)-2-specific drugs, a great interest has been applied to nutraceutical supplements, which include a heterogeneous class of molecules with great potential to reduce inflammation, oxidative stress, pain, and joint stiffness and improve cartilage formation. The purpose of this review is to describe the potential application of nutraceuticals in OA, highlighting its molecular mechanisms of actions and data of efficacy and safety (when available).

## 1. Introduction

Osteoarthritis (OA) is a degenerative inflammatory condition of the joint cartilage that currently affects approximately 58 million adults, with an estimated increase to 78.4 million by 2040 [1]. It represents the principal reason for joint pain and functional impairment in the world [2].

This inflammation is characterized by pain, stiffness, and a reduced range of motion with regard to the arthritic joints. These symptoms can cause a greater risk of overweight/obesity, diabetes mellitus, and falls and fractures in the long term [3]. Factors predisposing to OA could be classified as local biochemical factors, including joint injury, joint space, and physical activities, and general factors, such as sex, age, comorbidities like obesity, and nutrition disorders [4].

Current guidelines for the treatment of OA suggest three types of approaches, which can be also combined if necessary. The first approach includes pharmacological treatment, which is characterized by non-steroidal anti-inflammatory drugs (NSAIDs), opioids, and cyclooxygenase (COX)-2-specific drugs. However, it has only a “palliative” role by reducing symptoms but not considering the essential problem of the cartilage disorder. In addition, the conventional therapies can cause (especially for long period of consumption) possible side effects, which can reduce the compliance for the appearance of gastrointestinal problems, cardiovascular effects, and others [5]. The second approach regards lifestyle change, a non-pharmacological approach characterized by rehabilitation to facilitate healthy body composition, physical activity, and the optimization of an appropriate nutrition plan and a nutraceutical treatment [6]. In this context, a chronic nutritional intervention associated with conventional therapies demonstrated to improve OA condition (joint of knee, hip, and hand) compared with only pharmacological treatments [7]. If lifestyle changes and medications are not enough, the third approach is surgery. Nevertheless, in recent years, a great interest has been applied to nutraceutical supplements, which include a heterogeneous class of molecules with great potential to reduce inflammation, oxidative stress, pain, and joint stiffness and improve cartilage formation [8]. The use of nutraceuticals might be particularly interesting for the action on pain, which is typically chronic in OA and represents the main cause of disability for this condition. In this regard, for evaluation of OA pain intensity, the most common criteria include the visual analog scale (VAS) or numerical rating scale (NRS), the pain subscale of the Western Ontario and McMaster Universities Arthritis Index (WOMAC), and the Knee injury and Osteoarthritis Outcome Score (KOOS) [9]. In particular, the WOMAC index is widely used in the evaluation of hip and knee osteoarthritis based on a self-administered questionnaire consisting of items regarding pain, stiffness, and physical function. In this regard, a recent meta-analysis of 42 random clinical trials (RCTs) utilizing nutraceuticals (such as chondroitin sulphate, glucosamine sulphate, collagen, and hyaluronic acid) found improvements across all OA measurement parameters expressed through the total WOMAC index, WOMAC pain and WOMAC stiffness subscales,and VAS [10]. Among the most used nutraceuticals in OA, chondroitin sulphate, glucosamine sulphate, collagen, hyaluronic acid, and methylsulfonylmethane were shown to be impressive in the improvement of clinical symptoms and in decreasing inflammatory indices in subjects with OA [11]. Confirming this, 69% of patients with OA receive various forms of nutraceuticals for their problem [12]. However, although numerous studies have detailed the benefits of nutraceutical usage in OA, inconsistent reports of the side effects and results of little statistical significance have kept them from mainstream medical usage as described in further sections.

The purpose of this narrative review is to describe the molecular mechanisms of action of the most used and clinically tested chondroprotective nutraceuticals.

## 2. Methods

As a first step, we focused our literature search on the search for the most clinically studied dietary supplements using as key words on PubMed “Osteoarthritis”, “Nutraceuticals”, “Dietary supplements”, “Randomized”, and “Clinical trials”. Then, we searched for studies investigating the mechanism of action of the selected nutraceuticals and critically described it.

A summary of the discussed nutraceuticals and their proposed mechanisms of actions is reported in Table 1.

## 3. Nutraceuticals Used for the Prevention of Worsening and Management of OA

### 3.1. Glucosamine and Chondroitin

Chondroitin and glucosamine are nutraceuticals commonly used in clinical practice for OA patients for its analgesic and chondroprotective effects [33]. Glucosamine is a water-soluble amino monosaccharide available in two forms (glucosamine sulphate and glucosamine hydrochloride), which is a normal constituent of glycosaminoglycans (GAGs) in cartilage matrix and in the synovial fluid and consequently present in high quantities in articular cartilage. It is a constituent of keratan sulphate. Chondroitin is a major component of the extracellular matrix of articular cartilage, which played an important role in creating considerable osmotic pressure. In this way, it could provide cartilage with resistance and elasticity to resist tensile stresses during loading conditions [34].

#### 3.1.1. Mechanisms of Action

Glucosamine and chondroitin act first as anti-inflammatory agents. A study conducted in rats treated with different dosages of glucosamine (20, 40, 80, or 160 mg/kg/day) showed, by the 6th day, the capability of this substance to reduce the levels of proinflammatory cytokines interleukin (IL)-1, IL-6, and tumor necrosis factor (TNF)-α, also preventing the increase in plasma nitrite levels [35]. Similar results were obtained by Li et al., and Waly et al., on mice with monosodium iodoacetate-induced OA in which glucosamine was shown to downregulate serum pro-inflammatory cytokines (IL-1β, IL-6, and TNF-α) and C-reactive protein levels and upregulate the anti-inflammatory cytokines IL-2 and IL-10 levels [17,18]. Oral glucosamine administration is able to suppress the early increase in tumor grown factor (TGF)-β levels in monosodium iodoacetate-induced OA rats [17]. Nevertheless, the precise role of TGF-β in different stages of OA is still unclear as well as the effects of glucosamine on this function required further confirmations [17]. Glucosamine appears to also have immune-modulatory activity by inhibiting the expression and/or activity of catabolic enzymes, such as phospholipase A2, MMPs, or aggrecanases [36].

Both chondroitin and glucosamine sulphate were demonstrated to reduce the activation of the nuclear factor κB (NF-κB) and p38 mitogen-activated protein kinase (MAPK) pathway, which represent a pathway of inflammation in OA [37]. In addition, further studies showed that these molecules prevent the cytokine-induced IL-1β expression by suppressing the demethylation of the IL-1β promoter region [36], inhibiting the lipopolysaccharide (LPS)-induced reactive oxygen species (ROS) generation, nucleotide-binding oligomerization domain-like receptor containing pyrin domain 3 (NLRP3) inflammasome activation and caspase-1 activation, and upregulation and release of IL-1β [38]. 

Another important activity of both chondroitin and glucosamine concerns its antioxidant properties, which have been reported in several in vitro models [21]. Even though glucosamine hydrochloride and glucosamine sulphate possess antioxidant activity, glucosamine sulphate shows greater antioxidant and radical scavenging activities compared with glucosamine hydrochloride [39]. 

The reduction of ROS produced during the early OA stage by mechanical stress, trauma, or chemicals is important in order to reduce the cellular damage on the adjacent cartilage and collagen degradation [40]. Glucosamine was shown to bind directly with malondialdehyde (MDA) and block the subsequent formation of MDA adducts and protein cross-linkages and thus prevent lipoprotein oxidation and inhibit MDA adduct formation in the articular chondrocyte cell matrix [41]. Other in vitro studies reported the ability of these molecules to inhibit hydrogen peroxide (H2O2)-mediated membrane lipid peroxidation, protein and DNA oxidation, as well as the intracellular ROS level in chondrocytes, with the upregulation of glutathione (GSH) and other antioxidant enzymes, including superoxide dismutase (SOD), catalase (CAT), and glutathione peroxidase (GPx) in chondrocytes [42]. However, direct evidence of the antioxidant activity of both glucosamine and chondroitin in OA animal models is limited. In a study conducted in the formalin-induced osteoarthritic rat knee joint, the association of glucosamine hydrochloride, chondroitin sulphate, methylsulfonylmethane, *Harpagophytum procumbens* root extract, and bromelain administered orally for 30 days resulted in a significant reduction in MDA, NO, and 8-hydroxyguanine levels and an increased total GSH level was observed. These reductions were higher in the combination group (with plants) if compared with the administration of glucosamine or chondroitin alone, probably due to the additional analgesic effects [43].

Glucosamine and chondroitin are also known to improve tissue regeneration as demonstrated by different in vivo studies. In New Zealand rabbits with surgical-induced knee joint cartilage damage, treatment with glucosamine sulphate remarkably improved hyaline cartilage regeneration on autologous chondrocyte implantation repair sites, with upregulation of proteoglycans, type II collagen, and GAGs expression compared with autologous chondrocyte implantation alone [19]. In addition, these nutraceuticals simultaneously suppressed the osteoclastic cell differentiation in MC3T3-E1 osteoblasts, downregulating the receptor activator of NF-κB ligand (RANKL) expression [44] and blocked IL-1β-mediated downregulation of type II collagen and aggrecan gene expression and inhibited the MMP-13 gene expression in both normal and OA chondrocytes [45].

#### 3.1.2. Efficacy and Safety

Chondroitin and glucosamine were tested in several clinical trials of OA. However, the results remain at least in part contrasting. A recent meta-analysis of 30 randomized clinical trials including 7127 patients showed that chondroitin could alleviate pain symptoms (−0.071, 95%CI: −0.228 to 0.085) and improve physical function (−0.090, 95%CI: −0.242 to 0.061) compared with the placebo [46]. Similar results were shown in a previous meta-analysis in which treatments with glucosamine and chondroitin were found to significantly reduce pain in VAS [weighted mean difference (WMD) −7.41 mm, 95%CI–14.31, –0.51, *p* = 0.04 and WMD–8.35 mm, 95% CI–11.84, –4.85, *p* < 0.001, respectively] [47]. Even the meta-analysis by Ogata et al., including 18 RCTs, demonstrated glucosamine’s potential to alleviate knee OA pain [48]. Moreover, Zhu et al., reported the superiority of the combination of glucosamine and chondroitin for pain reduction and joint stiffness in comparison with acetaminophen, even if celecoxib was most likely the best option for knee or hip OA [49]. In a large meta-analysis of 54 randomized clinical trials including 16,427 patients, the effects of glucosamine and chondroitin were compared with conventional treatments. Although, even in this case, celecoxib was considered the best option for pain reduction, only glucosamine plus chondroitin showed a clinically significant improvement from baseline function. In terms of the structure-modifying effect, both glucosamine alone and chondroitin alone achieved a statistically significant reduction in joint space narrowing [50].

However, other studies reported no significant improvement of the OA condition after both glucosamine and chondroitin supplementation. Kwoh et al., did not observe any significant changes in the joint structure in people with chronic knee pain treated with glucosamine 1500 mg/day [51]. In addition, supplementation with chondroitin or glucosamine (1500 mg/day) for 2 years also did not cause remarkable changes in the joint structure of OA patients (aged 45–75 years) [52]. Similar conclusions were obtained from a study regarding 59 people with temporomandibular OA randomly receiving glucosamine sulphate 1200 mg/day or placebo. At the end of the study (six weeks), no significant differences in all signs and symptoms of OA were observed in both groups [53]. In accordance with this data, in a meta-analysis of 17 clinical studies, only seven showed a statistically significant reduction in pain (−0.35, 95%CI = −0.54 to −0.16) and four studies met the review criteria for joint space narrowing (SMD= −0.10, 95%CI = −0.23 to +0.04) [54]. Moreover, several smaller dosages throughout the day of glucosamine seem to be better in pain reduction effects if compared with a single daily large dose (1500 mg) [38].

Both glucosamine and chondroitin were also demonstrated to reduce joint inflammation and oxidative stress. In an RCT including elderly people with temporomandibular joint OA, treatment with an intra-articular hyaluronic acid injection in combination with glucosamine hydrochloride (oral 720 mg for 3 months) was shown to be greatly beneficial by further reducing the IL-6, IL-1β, and TGF-β levels if compared with people treated with only hyaluronic acid injection [55]. Glucosamine sulphate (1500 mg/day) in addition with celecoxib (200 mg/day) supplemented in patients with knee OA for 8 weeks showed a better redox status expressed as higher serum SOD activity and lower serum MDA levels compared with the control group (celecoxib alone) [56]. 

In conclusion, data indicates that both glucosamine and chondroitin may have a small to moderate effect in reducing OA-related pain and joint inflammation but little effect on joint-space narrowing. The discrepancies in the effectiveness of chondroitin and glucosamine on osteoarthritis between different studies should be further examined.

The supplementation of both chondroitin and glucosamine in the medium term is considered safe and well tolerated. Different studies reported some mild and transitory effects, such as diarrhea, abdominal pain, nausea, and headache. However, there was no significant difference in the comparison between any options (glucosamine alone, chondroitin alone, glucosamine + chondroitin) versus placebo. 

### 3.2. Methylsulfonylmethane

Methylsulfonylmethane (MSM) is a supplement containing organic sulphur suggested for relieving joint/muscle pain, thanks to its anti-inflammatory properties and it has also been reported to slow anatomical joint progressivity in the knee OA. It is a natural member of the methyl-S-methane compounds, but it can also be obtained through the oxidation of dimethylsophoxide with H_2_O_2_ [42]. 

#### 3.2.1. Mechanism of Action

In vitro studies showed the ability of MSM to act as an anti-inflammatory agent through the inhibition of the nuclear factor kappa-light-chain-enhancer of activated B cells (NF-κB) [57] as well as p65-S536 phosphorylation [58], reducing the expression of interleukins (IL-1, IL-6, IL-1β) and TNF-α [59,60]. MSM downregulates NF-κB production of the leucine-rich repeat family pyrin domain-containing 3 (NLRP3) inflammasome transcript and/or it blocks the activation signal in the form of mitochondrial-generated ROS, negatively affecting the expression of the NLRP3 inflammasome [44]. In addition, MSM reduces the expression of cyclooxygenase-2 and nitric oxide synthase, reducing the formation of superoxide radical (O_2_^−^) and nitric oxide (NO) [61]. MSM influences the balance of ROS and antioxidant enzymes by mediating at least four types of transcription factors including NF-κB, signal transducers and activators of transcription (STAT), p53, and nuclear factor (erythroid-derived 2)-like 2 (Nrf2) [45]. Other pleiotropic effects of MSM include the improvement of osteoblast differentiation acting through the Jak2/STAT5b pathway [62] and the upregulation of the master gene RUNX2, which transactivates its downstream partner SP7 (Osterix) and thus the expression of osteogenic genes (e.g., COL1A1 and COL1A2 (collagen type I), BGLAP (osteocalcin), SPARC (osteonectin), and SSP1 (osteopontin)) [63]. The osteogenic differentiation operated by MSM is also stimulated by the induction of BMP2 or transglutaminase-2 (TG2) [64]. 

#### 3.2.2. Efficacy and Safety

Although the MSM anti-arthritic data of in vitro and in animal models are numerous and promising [58,65], human studies are for the most part conducted on small samples of people and in combination with other molecules, such as chondroitin and glucosamine.

RCTs suggest MSM is effective in reducing pain, as indicated by the WOMAC pain subscale [66,67], the 36-Item Short Form Survey (SF36) pain subscale [26], the VAS pain scale, and the Lequesne Index [68]. Significant improvements were also observed in swelling [55] and stiffness [53].

Other RCTs utilizing MSM in combination with other therapies report similar results. In this regard, the study conducted by Usha et al., reported an improvement in pain, pain intensity, and swelling after supplementation with MSM and glucosamine, in people with OA [55]. In another double-blind RCT including 147 people with knee OA, Kellgren–Lawrence grade I-II, patients were randomized to receive 1500 mg of glucosamine + 1200 mg of chondroitin sulphate or 1500 mg of glucosamine + 1200 mg of chondroitin sulphate + 500 mg of MSM or placebo for 3 months. At the end of the treatments, the MSM treatment group was found to have a statistically significant WOMAC score (*p* = 0.01) and VAS score (*p* < 0.001) compared with the control and the glucosamine and chondroitin group (WOMAC score (*p* = 0.03) and VAS score (*p* = 0.004) [69]. The combination of glucosamine, chondroitin, and MSM has also been demonstrated to improve functional ability and joint mobility [70]. However, these positive observations were not confirmed in the trial by Magrans-Courtney et al. [71]. Further larger and long-term clinical trials are needed to quantify its efficacy, especially when compared to chondroitin and glucosamine.

MSM appears to be well-tolerated and safe as reported by the Food and Drug Administration (FDA) GRAS notification, at dosages under 4845.6 mg/day [72]. 

### 3.3. Collagen

Collagen is an extracellular matrix protein localized in the skin, tendons, cartilage, and bone, which is usually contained in foods, such as fish and meat [73]. It is characterized by a triple-helix configuration with a repeating amino acid sequence, (Gly-X-Y)n, where X and Y are typically proline and hydroxyproline, respectively. Type II collagen is the major component (90–95% of total collagen content) of the articular cartilage, forming the fibrils that give cartilage its tensile strength [74]. It is also generally commercialized as a nutritional supplement obtained by the action of proteases, which hydrolyze the gelatine [75]. In fact, the bioavailability of undenatured collagen is very low because it is not hydrolyzed and thus physiologically available for enteric absorption [76]. However, it has been demonstrated that bioactive di- or tripeptides containing proline and hydroxyproline could be absorbed into the blood circulation and exert anti-OA activities [77]. 

#### 3.3.1. Mechanism of Action

The site of interaction of collagen and its subfractions on cartilage has not yet been fully clarified. Several in vitro studies have investigated the potential molecular mechanisms regarding the protection of articular cartilage [78]. Firstly, collagen is well known to be an extracellular matrix molecule used by cells for structural integrity [79]. Hydrolyzed collagen may induce cartilage regeneration by increasing the synthesis of macromolecules in the extracellular matrix [80]. After a single administration with labeled collagen peptide, a significant improvement in the levels of collagen detected in the skin and cartilage was shown, indicating an accumulation of these peptides in the connective tissue [81]. 

Collagen is able to modulate the humoral and cellular immune response. In fact, it activates the immune cells (it takes place in the gut-associated lymphoid tissue (GALT)) and regulates the oral tolerance, which is an immune process that the body uses to distinguish between innocuous molecules, such as dietary proteins or intestinal bacteria, from potentially harmful foreign invaders [82]. In this regard, the transformation of naive T cells into T regulatory (Treg) cells permits the secretion of anti-inflammatory mediators, including the transforming growth factor-beta (TGF-β), interleukin 4 (IL-4), and interleukin 10 (IL-10), which help in the reduction of joint inflammation and promotion of cartilage repair [83,84].

Proline and hydroxyproline are considered to be the biologically active substances of collagen, inducing hyaluronic acid synthesis as reported by in-cultured synovial cells [85] and suppressing the hypertrophic differentiation of chondrocytes, which is well known to be involved in the pathogenesis of OA [86]. Furthermore, these amino acids stimulate glycosaminoglycan synthesis by chondrocytes [79]. In a rat experimental osteoarthritis (OA) model, collagen peptides administration was demonstrated to reduce the CTX-II (type II collagen degradation) levels and suppress the Mankin score (*p* < 0.05 for both). In addition, type II collagen reduced inflammation expressed as MMP-13 levels [87].

Based on these findings, oral administration of hydrolyzed collagen has anti-inflammatory and chondroprotective actions, which could represent the basis of the protection of OA initiation/progression. Further in vitro studies investigating the components of collagen peptides are required to elucidate the detailed mechanism behind the beneficial effect of these peptides on joint health.

#### 3.3.2. Efficacy and Safety

Different clinical studies have investigated the protective effects of collagen on joints [88,89]. It has been suggested that supplementation with 4.5–10 g/day of collagen peptides, at least for 2 months, relieves knee and hip joint pain in people with OA [90]. In a RCT involving 147 athletes, 10 g collagen/day for 24 weeks reduced activity-related joint pain [91]. In another RCT including 30 subjects with knee OA, supplementation of 5 g/day of collagen for 13 weeks showed a significant improvement of all the score levels of WOMAC, VAS, and quality of life (*p* < 0.01 compared with placebo). In accordance with these results, in a meta-analysis of five RCTs, collagen treatment showed a significant reduction in the total WOMAC index score (WMD–8.00; 95% CI–13.04, –2.95; *p* = 0.002) and in the VAS score (WMD–16.57; 95% CI–26.24, –6.89; *p* < 0.001). Subgroup analysis of the WOMAC subscores also revealed a significant decrease in the stiffness subscore (WMD–0.41; 95% CI–0.74, –0.08; *p* = 0.01) [20]. Based on the available data, it is estimated that collagen peptides are effective at dosages of 0.166 g/kg body weight/day (10 g/60 kg/day) in treating joint pain in humans [92]. In the multicenter RCT by Trc et al., supplementation for 90 days of hydrolyzed collagen at a dosage of 10 g/day was demonstrated to be superior to glucosamine sulphate 1.5 g/day in improving the WOMAC and VAS score (reduction of the WOMAC and VAS index observed in the collagen group: 80.8% of the study population vs. 46.6% observed in the glucosamine sulphate group) [93]. The pain reduction after collagen supplementation in patients with OA indirectly indicates an improvement in joint conditions. In fact, as discussed below, the initiation of the repair process by the accumulation of collagen peptide in cartilage tissue helps to maintain the structure and function of cartilage, resulting in joint comfort, subsequent improvement in pain, and a reduction in the progression of degradation of articular cartilage tissue.

Collagen supplementation is generally safe with no reported adverse events. Further studies are needed to elucidate medical use in OA disease and to determine the optimal dosing regimens as well as the period of treatments.

### 3.4. Hyaluronic Acid

Hyaluronic acid (HA) is a mucopolysaccharide constituted by repeated monomers of β-1,4-D-glucuronic acid and β-1,3-*N*-acetylglucosamine. This molecule is particularly present in the synovial fluid with excellent viscoelasticity, high moisture retention capacity, high biocompatibility, and hygroscopic properties, thus acting as a lubricant, shock absorber, joint structure stabilizer, and water balance and flow resistance regulator [94]. HA is considered the treatment of choice for people with knee/hip OA, working slowly if compared with steroid treatments, but its effect may last considerably longer [95]. Although HA injection has shown great advantages in improving the clinical symptoms of OA patients, it must be administered repeatedly into the joint cavity [96]. The need for multiple injections of HA is a major drawback of the therapy because of the proportional increase of side effects with the repeated injections and the discomfort of repeated clinic visits. For these reasons, considering the disadvantages of HA injection, it is more favorable for the symptoms of OA to be relieved by oral administration [97].

#### 3.4.1. Mechanism of Action

The first proposed HA mechanism of action regards chondroprotection. HA has been shown to reduce chondrocyte apoptosis, while increasing chondrocyte proliferation [98]. This phenomenon is possible through HA binding to cluster of differentiation 44 (CD44) receptors that cause inhibition of interleukin (IL)-1β expression and a decline in matrix metalloproteinase (MMP)−1, 2, 3, 9, and 13 production [99]. HA also binds to the receptor for hyaluronan-mediated motility (RHAMM), which is thought to aid in chondroprotection in addition to CD44 binding [92]. In addition, it may affect the subchondral bone by the suppression of MMP-13 and IL-6 via CD44 binding [100]. The suppression of MMP-13 expression has been suggested to be a critical factor in the effect on OA subchondral bone [93]. 

Although human studies demonstrated that oral supplementation with HA can reach the blood and be distributed to the skin and joints [101], the therapeutic effects of this molecule on OA may not necessarily require its absorption. HA could in fact bind to toll-like receptor 4 (TLR4) in the intestine and exert its biological activities, increasing the secretion of suppressor of cytokine signaling 3 (SOCS3), which leads to the suppression of pro-inflammatory cytokine expression [102]. In this regard, the suppression of pro-inflammatory mediators IL-8, IL-6, PGE2, and TNFα has been observed with HA [103]. In addition, the binding of HA to TLR4 also suppresses the expression of pleiotrophin, a pro-inflammatory molecule [102]. HA also binds to the intercellular adhesion molecule (ICAM-1), downregulating NF-kB, which in turn decreases the production of IL-6 [104]. 

Another possible mechanism of action regards the N-acetyl glucosamine, which is the monosaccharide that comprises HA together with D-glucuronic acid. N-acetyl glucosamine is converted into glucosamine in the cells by the actions of lysosomal enzymes, which permit the chondroprotective and anti-inflammatory activities of glucosamine described in the dedicated paragraph [105]. HA treatment stimulated proteoglycan synthesis (such as aggrecan), reducing the progression of OA [106]. 

HA has a mechanical effect, which contributes to the lubrification of the joint capsule, preventing degeneration through decreased friction. HA provides cushioning to absorb pressure and vibration, avoiding the degradation of chondrocyte tissue [107].

HA possesses analgesic effects, probably by decreasing the mechanical sensitivity of stretch-activated ion channels, which effectively blocks the pain response. Moreover, HA reduces the action of joint-sensitized nociceptors, which are affected by the HA concentration, reducing the pain response exhibited by these terminals [108].

#### 3.4.2. Efficacy and Safety

The results of animal experiments using radiolabeled HA clearly demonstrated that oral HA would indeed be absorbed and distributed to the skin, bone, and synovial joints and would be retained in those tissues for prolonged periods [109]. In agreement with these findings, RCTs in humans showed that oral supplementation with this molecule can reach the blood and can be distributed to the skin and joints, preserving its biological activities [101].

Several studies have suggested that oral supplementation with HA could alleviate the symptoms of knee OA [110,111]. Sato et al., demonstrated that treatment with 240 mg/day of HA for 12 weeks was associated with an improvement of the Japanese Knee Osteoarthritis Measure (JKOM) score and the Japan Orthopaedic Association (JOA) score improved significantly compared with baseline values [112]. Supplementation with 200 mg/day of HA in OA people for 8 weeks was also shown to improve the WOMAC scores compared with the placebo group [108]. Similar results were described by Iwaso et al., who highlighted an improvement of both WOMAC and JKOM scores after 200 mg of HA supplementation for 8 weeks [113].

In a one-year RCT, 60 osteoarthritic subjects (Kellgren-Lawrence grade 2 or 3) were randomly assigned to receive HA (200 mg/day) or placebo. At the end of treatment, the improvement was most evident in the subscale for “pain and stiffness”, and it was more obvious in patients who performed therapeutic exercises in combination with HA therapy [114]. 

Although different studies report positive effects of oral HA supplementation on OA, several limitations should be described before arriving at any conclusion. In fact, the lack of appropriate controls, shortness of study periods, small sample size, as well as the different forms of supplemented hyaluronic acid (in many cases, this was not described in the studies) may influence the results. To clarify these issues, new long-term RCTs with standardized HA are urgently required and desirable to recommend such a nutraceutical in clinical practice.

It was confirmed that oral supplementation of HA is safe in humans at dosages up to 200 mg/day ingested for periods up to 12 months [109]. Several safety studies were conducted with medium-term (3–6 months) supplementation periods, and safety was confirmed [115]. 

### 3.5. Vitamin C

Vitamin C or ascorbic acid was first isolated in 1923 by Szent-Gyorgyi and synthesized by Howarth and Hirst. It is especially found in citrus fruits, red and green peppers, tomatoes, strawberries, broccoli, Brussels sprouts, turnips, and other leafy vegetables. It is a water-soluble molecule with highly effective antioxidant properties due to its reactivity with numerous aqueous-free radicals and ROS [116]. A deficiency of this molecule can be associated with a higher risk of various diseases including scurvy, anemia, capillary hemorrhage, muscle degeneration, infections, bleeding gums, poor wound healing, atherosclerotic plaques, and neurotic disturbances. In addition, dietary intake of vitamin C seems to be associated with a decreased risk of cartilage loss and OA in humans, probably by the reduction of oxidative stress, as highlighted by different studies [117,118]. However, although vitamin C has an approved claim by the EFSA (European Food Safety Authority) regarding its role on the normal formation of collagen, it is still unknown whether this supplement has additional effects on the prevention of OA progression.

#### 3.5.1. Mechanism of Action

Vitamin C acts prevalently as an antioxidant and antiradical supplement. In fact, it exists in both reduced (ascorbate) and oxidized forms as dehydroascorbic acid, which are easily inter-convertible and biologically active, thus it acts as an important antioxidant. In this regard, ascorbic acid reacts directly with free radicals undergoing single-electron oxidation to produce a relatively poor reactive intermediate, the ascorbyl radical, which disproportionates to ascorbate and dehydroascorbate. In this sense, ascorbic acid has the ability to reduce toxic ROS superoxide anion (O^2^•), and hydroxyl radical (OH•), as well as organic (RO^2^) and nitrogen (NO^2^•) oxy radicals. In addition, this molecule could also act indirectly, by protecting the oxidation of other vitamins, such as vitamin A and E. In this context, damage caused by ROS has long been thought to be pathogenic and it has an important role in the progression of OA, causing cytotoxicity and cellular damage [8]. Although OA is classified as a ‘non-inflammatory arthropathy’, growing evidence highlights the key role of pro-inflammatory mediators in the pathogenesis of OA [119]. Vitamin C also plays an important role as an electron donor in the synthesis of type II collagen [120] and a sulphate carrier in glycosaminoglycan synthesis [121]. Moreover, ascorbic acid is a co-factor for the hydroxylation and activity of mono-oxygenase enzymes in the synthesis of collagen, carnitine, and neurotransmitters, maintaining the active center of metal ions in a reduced state for optimal activity of the enzymes hydroxylase and oxygenase [109]. The depletion of sulphated proteoglycans’ extracellular matrix of the articular cartilage is considered one of the most relevant expressions of OA, often associated with cartilage degeneration [117]. Vitamin C deficiency could therefore be considered a risk factor for OA development and thus, its supplementation in primary prevention may represent a possible solution for OA, especially in combination with conventional or unconventional therapies. 

#### 3.5.2. Efficacy and Safety

Vitamin C possesses multiple abilities for the prevention of OA progress, including the modulation of apoptosis processes (via procaspase-3 and procaspase-9 and Bax expression) and the expression of pro-inflammatory cytokines and MMPs, in addition to the well-known antioxidation. In an in vitro chondrosarcoma cell line (SW1353), treatment with 100 µM of vitamin C was shown to prevent oxidative stress, apoptosis, and proteoglycan loss induced by the treatment with 5 µM of monosodium iodoacetate (MIA). Moreover, the expression levels of the pro-inflammatory cytokines IL-6, IL-17A, and TNF-α and MMP-1, MMP-3, and MMP-13 were also prevented [122]. Similar results were obtained in in vivo monosodium iodoacetate (MIA)-induced OA rats (vitamin C supplemented: 100 mg/kg) [118]. Many in vivo studies indicated that increased intake of vitamin C may decrease the risk of OA progression and cartilage loss in humans, a causal association with its capacity against oxidative stress [113,117]. However, some studies indicated a non-correlation between circulating vitamin C levels and incident radiographic knee OA [123]. In a prospective cohort study including 1023 people, individuals without baseline knee OA who self-reported vitamin C supplement usage were 11% less likely to develop knee OA than those individuals who self-reported no vitamin C supplement usage (risk ratio (RR)=0.89, 95% CI 0.85, 0.93). In addition, among those participants with radiographic knee OA at baseline, after controlling for confounding variables, vitamin C supplementation was demonstrated to be potentially useful in preventing incident knee OA [124]. In a cross-sectional analysis (4685 participants) regarding the associations between the intake of dietary antioxidants (carotenoid, vitamin C, E, and selenium) and radiographic knee OA, radiographic knee OA was not significantly associated with dietary carotenoids, vitamin E, and selenium but significantly associated with vitamin C intake [117]. Moreover, the use of vitamin C significantly decreased the lipoperoxides (*p* < 0.05 compared with the placebo group) linked with hip bone loss [125]. Vitamin C may also reduce the use of painkillers and improve quality of life, probably acting as a cofactor for the enzyme peptidyl glycine α-amidating mono-oxygenase (PAM), which is involved in the synthesis of the endomorphin-1 [30,126].

Given the massive economic burden of OA, the use of a simple, inexpensive, and widely available supplement to potentially reduce the impact of this disease merits further consideration. However, long-term RCTs are urgently required before making any final consideration. 

Overall, vitamin C appears to be a safe and effective adjunctive therapy for acute and chronic pain relief in specific patient groups [125]. Nevertheless, future studies are needed in order to measure the vitamin C concentrations at baseline and following intervention to determine if specific patient groups respond, determination of the optimal route of administration, the optimal dose and frequency of administration, and the potential mechanisms of action of this molecule in OA disease.

### 3.6. Vitamin D 

Vitamin D is well known to be a lipophilic molecule, which was recommended by the Institute of Medicine (IOM) in 2011for bone health, in order to improve the calcium absorption, bone mineral density, and vitamin D deficiency rickets/osteomalacia [127]. Although it can be obtained both through foods, such as mushrooms fatty fish, and vitamin D-fortified products, and through the cutaneous synthesis in response to ultraviolet-B exposure, vitamin D deficiency occurs in <20% of the population in northern Europe; in 30–60% in western, southern, and eastern Europe; and up to 80% in Middle East countries. Severe deficiency (serum 25(OH)D < 30 nmol/L or 12 ng/mL) is found in >10% of Europeans [128].

Several studies have investigated the possible role of vitamin D on the initiation and progression of OA. In this regard, the expression of vitamin D receptors (VDRs) in the articular cartilage of OA people, but not in that of healthy volunteers, may indicate the direct influence of this hormone on the potential damage of articular cartilage [129,130]. In addition, vitamin D may also act on OA indirectly through the endocrine system. 

#### 3.6.1. Mechanism of Action

Vitamin D is an important immunoregulator of inflammation, influencing the response of white cells (macrophages, dendritic cells, T and B lymphocytes) [131]. In particular, the activation of VDR localized on white cell membranes also promotes the blocking of cytokine genes’ transcription, such as NF-AT (nuclear factor of activated T cells) and NF-κB, reducing the production of TNF-α and IL-1, which are considered inflammatory pathways for the deterioration of cartilage [129]. In addition, several studies have shown that VDRs are upregulated on damaged cartilage. An in vitro study reported the hyperexpression of VDR in the areas of erosion of late-stage rheumatoid arthritis cells [129]. This result was confirmed in humans by the same research group [131] and by Orfanidou et al. [132]. The upregulation of VDR seems to stimulate a signaling cascade that enhances the production of MMPs 1, 3, and 9 from chondrocytes, which induces the degradation of both cartilage and bone [133].

Vitamin D acts also with the VDRs expressed on osteoblasts. A study by Corrado et al. [134] found that OA osteoblasts expressed a significantly lower receptor activator of NF-*κ* B ligand (RANKL)/osteoprotegerin (OPG) ratio compared to healthy and osteoporotic cells. RANKL and its decoy receptor OPG are regulators of osteoclastogenesis and thus bone resorption. Moreover, OPG expression in OA osteoblasts was significantly higher than in controls and in osteoporosis patients. Nevertheless, these results contrasted with those obtained by Giner et al., who found OPG secretions to be higher in osteoporotic osteoblasts compared to OA [135]. 

Vitamin D may regulate the angiogenesis process, which is an important pathway in the pathophysiology of OA. In fact, the expression of vascular endothelial growth factor (VEGF) is regulated in part by 1*α*,25(OH)2D3 as demonstrated by studies in vitro, including osteoarthritic osteoblasts [136]. This supports the idea that osteoblasts could regulate angiogenesis in subchondral bone and may link vitamin D with the development and progression of OA.

Limited evidence suggests that vitamin D acts indirectly on osteoclasts through the activation of the abovementioned RANKL signaling, increasing bone resorption [137].

Lastly, vitamin D may also regulate the transforming growth factor-beta (TGF-β)/SMAD pathway that is involved in OA [133]. In healthy joints, TGF-β protects the joint, repressing MMP13 expression [138]. In this regard, the blockage of TGF-β, the expression of which decreases with age, results in alteration of the repairing responses in the joint and cartilage damage [139]. However, in contrast with these results, TGF-β seems to aggravate OA in OA joints. The inhibition of TGF-β signaling was shown to attenuate OA in the mesenchymal stem cells of subchondral bone [140]. These observations indicate that TGF-β may protect healthy joints from OA, even if it may aggravate the condition of joints with existing OA. Nevertheless, vitamin D should be recommended, especially in OA elderly with low plasmatic levels of this hormone (<30 ng/mL) associated with comorbidities, such as cardiovascular diseases and disorders related to bone health. In fact, it was demonstrated to reduce oxidative protein damage, decrease pain (VAS), improve quality of life, and improve grip strength and physical performance in these patients [141].

#### 3.6.2. Efficacy and Safety

Whether vitamin D deficiency increases the risk of developing OA is still unclear and results are contrasting. Several observational studies have shown no relationship between relatively high (≥50 nmol/L) vitamin D status and OA initiation expressed as pain, radiologic OA, and cartilage volume loss [142,143,144,145]. In a study in Ireland including rheumatology outpatients, 26% were found to be severely deficient (<12 ng/mL) and 70% vitamin D deficient (<21 ng/mL) [146]. In this regard, 62% of OA patients suffered from hypovitaminosis D and a low vitamin D status has also been associated with radiographic hip OA [147]. Similar conclusions were obtained by the osteoporotic fractures in a study of men in which vitamin D insufficiency or deficiency status were associated with a doubled risk of hip OA [148]. Even in Egypt [149] and in Iran [150], people with lower serum 25(OH)D3 had a major risk of developing knee OA. A systematic review by Cao and colleagues [151], including 15 studies, showed strong evidence for an association between 25(OH)D3 and cartilage loss in knee joints and moderate evidence to support a positive association between low levels of vitamin D and radiographic knee OA. In accordance with this observation, the Tasmanian Older Adult Cohort Study found an inverse correlation between time of sunlight exposure and knee cartilage loss [143].

In contrast with these results, the research group of Konstari and colleagues showed no correlation between vitamin D status and the risk of developing knee OA in Scandinavian people [152,153]. Felson et al., found no association between low vitamin D and structural worsening of affected joints (cartilage loss by magnetic resonance imaging and joint space narrowing by radiography) in two longitudinal studies (1203 people) [145].

People with deficiencies of plasma vitamin D (12.5–25 nmol/L) at baseline predicted a five-year change in knee pain and hip pain [154]. However, although elevated vitamin D status may attenuate joint pain and possibly radiologic OA in subjects with hypovitaminosis D [155,156], studies regarding the potential relationship between vitamin D status and the initiation of radiologic OA or cartilage volume loss in people with ‘suboptimal’ 25(OH)D are still lacking or contrasting. In a 2-year RCT, supplementation of cholecalciferol in patients with symptomatic knee OA showed no difference in the WOMAC knee pain scores or cartilage loss compared with the placebo group [156]. In partial accordance with this result, 787 members of the Hertfordshire Cohort Study (enrolled in a cross-sectional study) showed no association between vitamin D status and radiographic knee OA, even if there was a significant correlation between vitamin D and knee pain [157]. Furthermore, an RCT including 103 knee OA people treated with vitamin D oral supplements (60,000 IU/day for 10 days followed by 60,000 IU/month for 12 months) showed a small but significant correlation between the supplementation and the severity of pain and the functional scores (in comparison with the placebo group) [32]. 

In people undergoing total hip replacement, vitamin D levels have been shown to predict the OA outcomes, also suggesting a positive correlation of both preoperative and postoperative Harris hip scores and plasma 25(OH)D3 levels [158]. 

In conclusion, the research presented to date is conflicting as to the effects low vitamin D levels have on the functional aspects of knee OA. Although it appears that vitamin D has negative effects on OA cartilage health, as reported by the studies in vitro, which investigated the molecular mechanisms of action of this hormone, other human studies reported a possible role in prevention of pain, radiologic OA, and cartilage volume loss. In this context, to fully determine the relationship between vitamin D supplementation and the progression or development of OA, large scale longitudinal and multicentric studies including individuals with various skin pigmentations and different nationalities and ethnicities are urgent. In addition to this, large scale meta-analyses will yield more powerful interpretations of the existing findings and help to elucidate any associations low vitamin D has with OA.

Vitamin D supplementation is in general safe and well tolerated. The upper limit of vitamin D dose safety could differ depending on several factors, such as the vitamin D plasmatic levels, dose of administration and regimen, outcomes, as well as sex and age, which may play a role. However, the prevention or correction of vitamin D deficiency/insufficiency with 1000–2000 IU/daily of vitamin D is considered safe [159]. 

### 3.7. Other Promising Nutraceuticals

Avocado/soybean unsaponifiables have been shown to be effective in pain reduction as highlighted by human RCTs. In a 3-month RCT, daily intake of avocado/soybean unsaponifiables was demonstrated to significantly reduce the NSAID intake and the Lesquesne pain score [14]. Similar results were observed using the combination of avocado/soybean unsaponifiables with chondroitin sulphate (50% decrease in WOMAC scores) [160].

*Boswellia serrata* is well known to be rich in anti-inflammatory boswellic acids (3-O-acetyl-11-keto-beta-boswellic acid (AKBA), as the major potential active ingredient), which were shown to decrease pain (VAS, WOMAC pain), increase knee/hip flexion and walking distance (function and stiffness), as well as affecting the frequency of swelling if administered in patients with OA [5,161,162]. Similar results were observed in patients who took N-acetylglucosamine, ginger (5% gingerols at 250 mg/tablet), and *B. serrata* (65% boswellic acids at 180 mg/tablet), who reported a significant improvement in pain-free walking distance [15].

Another anti-inflammatory molecule is capsaicin, which is an agonist that binds to transient receptor potential V1 (TRPV1) channels in sensory nerve endings. This molecule extracted from the chili pepper is responsible for producing the sensation of heat or piquancy, which was shown to reduce mean WOMAC pain, stiffness, and functional scores compared with placebo in people with OA [16].

Curcumin, the most important curcuminoid in turmeric (*Curcuma longa*), has been well studied in OA disease, showing chondroprotective, antioxidative, and anti-inflammatory effects. In a 6-week trial including people with OA, the analgesic effect of oral curcumin (2 g/day) was comparable to ibuprofen (800 mg/day), improving the time spent walking 100 m and going up and down stairs [163]. The same conclusion was reported in another study in which curcumin (1500 mg/day) administered for 4 weeks resulted in a reduction in pain comparable to that with ibuprofen at 1200 mg per day [21]. In addition, it decreases inflammatory markers in the serum including IL-1β, IL-6, soluble CD40 ligand, sVCAM-1, and Coll2-1, a biomarker of type II collagen degradation [164]. 

Even ginger powder (titrated in gingerols) supplementation (1 g/day) can reduce inflammatory markers in patients with OA in addition to decreasing pain on standing, pain after walking, and WOMAC stiffness scores [23,24].

Polyphenols from pomegranate juice, pine bark, and green tea have demonstrated anti-inflammatory and antioxidative efficacy in relieving OA pain in human trials [13]. In particular, epigallocatechin 3-gallate (EGCG) from green tea reported a lower VAS pain score, as well as the total WOMAC score and physical function subscore after 4 weeks of administration in people with OA, compared with diclofenac 100 mg [22]. Similar results were observed with 150/200 mg/day of pycnogenol [29] and anthocyanins from pomegranate juice [13].

Lastly, omega-3 fatty acids (EPA and DHA) were reported to improve WOMAC pain, stiffness, and physical function scores and reduce NSAID use after 3 months of treatment [27]. These actions were confirmed even in association with the co-administration of 500 mg/day of glucosamine sulphate and other nutraceutical supplements like *Urtica dioica*, zinc, and vitamin E [165].

## 4. Discussion

Nutraceuticals supplementation has been shown to be a significant adjuvant strategy in the management of OA as reported by extensive evidence collected in cellular OA models, animals, and human RCTs.

From a molecular point of view, the abovementioned nutraceuticals act predominantly in the signaling pathways underlying OA pathogenesis, inflammation, and oxidative stress, with the former triggering the latter and vice versa [166]. 

Although complete knowledge of all the molecular effects in OA management is still lacking, a summary of the latter is shown in Table 1. As reported by different in vitro and in vivo studies, the advantage of most of the nutraceutical molecules is they exert pleiotropic effects, acting through different but complementary pathways of action regarding the reduction of inflammation and oxidative stress. For this reason, the synergistic integration of nutraceuticals with conventional therapy could eventually reduce the dosages and/or the number of administrations of drugs and thus the side effects [167].

An important point with regard to the nutritional supplements is the low bioavailability of the more promising compounds, which inevitably reduces the final effectiveness of the treatments. In this context, it is important to underline how the success of a nutraceutical supplementation does not depend exclusively on the correct choice of the active ingredient and the dosage of administration, but also on the correct bionutraceutical formulations [164]. This aspect may explain in part the great heterogeneity of results from clinical studies, which depend on the principles of clinical research, including the careful selection and stratification of the patients with inclusion and exclusion criteria, power analyses, and avoidance of confounding variables, in order to avoid inconsistent results.

However, one of the most relevant limits in the use of anti-OA nutraceuticals regards the high costs involved in obtaining titrated and standardized extracts. In general, the conventional techniques that are naturally available and used by industries do not always allow the production of inexpensive quality extracts and thus low-cost strategies to prevent or treat OA disease. The problem of cheap but poor-quality nutraceutical extracts is a serious, underestimated, and potentially dangerous problem for people’s health, not only because of the absence of specific active ingredients or the low quality of un-titrated extracts, but above all due to the presence of contaminants [168]. An important challenge for the future is to develop a food-compatible method for the extraction of specific phytochemicals and thus produce qualitatively effective and safe nutraceutical extracts with anti-OA properties. In this context, unconventional techniques, such as ultrasound or microwave-assisted extraction associated with hydroalcoholic solvents, may potentially improve the quality of the extracts and ensure good yields [169]. Nevertheless, although the unconventional processes used for extracting value-added products are well established in the laboratory, industrial-scale production with specific cost-effective analyses is still a challenge. 

Finally, another important goal in OA management is the reduction of pain, which remains a major source of distress and disability, and the improvement of quality of life. In fact, current pharmacological analgesics have limited efficacy, in addition to being potentially correlated with important adverse events, especially for chronic administrations. In this regard, the abovementioned nutraceuticals could be associated with other extracts of both botanical and animal origin in order to act comprehensively on the clinical picture of the patient with OA. While the specific molecular mechanism underlying the effect of these nutraceuticals on OA pain is at least in part unknown and not well understood, growing evidence suggests it may be attributable to their anti-inflammatory actions [11]. 

## 5. Conclusions

In conclusion, nutraceuticals may represent a valid strategy in OA management in association with conventional therapies. However, larger and longer studies are urgently required to definitively consider this unconventional approach in clinical practice, in order to focus the attention on the molecular mechanisms of nutraceuticals in mitigating OA processes and pain, the potential combination of anti-OA molecules with pain relief nutraceuticals, as well as both the efficacy and the safety of these treatments in long-term periods.

## Figures and Tables

**Table 1:** Nutraceuticals useful in OA diseases: dosages of administration, molecular mechanism of actions, and effect on OA-related behavior and biomarkers.

Nutraceutical	Tested Daily Dose	Molecular Mechanism of Actions	Effect on OA and Related Behavior	Ref.
Anthocyanins from pomegranate juice	300–600 mg	IL-1β, TNF-α, CCR2, NF-κB, JNK-MAPK, ROS, NO, COX-2, PGE2	↓ VAS pain, WOMAC pain	[13]
Avocado/soybean unsaponifiables	300 mg	IL-1, IL-6, IL-8, and PGE2	↓ VAS pain, WOMAC pain, ↓ Analgesic and NSAIDs use	[14]
Acetyl-keto-β-boswellic acid (AKBA) (from *Boswellia serrata)*	150–250 mg (AKBA)	iNOS, NF-κB, COX, LOX	↓ VAS pain, WOMAC pain	[15]
Capsaicin (from Chili pepper)	10 mg	TRPV1 agonist	↓ VAS pain, WOMAC pain	[16]
Chondroitin	500–1500 mg	IL-1, IL-6, TNF-α, IL-1β, TGF-β MMPs,NF-κB, ROS formation, improve of proteoglycans, type II collagen and GAGs expression	↓ VAS pain, WOMAC pain, stiffness, function and total, ↓ Analgesic and NSAIDs use	[17,18,19]
Collagen	4000–10000 mg	Cartilage regeneration by increasing the synthesis of macromolecules in the extracellular matrix, CTXII, MMP-13, T regulatory (Treg) modulation	↓ VAS pain, WOMAC pain, stiffness, function and total	[20]
Curcumin (from *Curcuma longa*)	1000–3000 mg	IL-1β, TNF-α, NF-κB, COX-2, PGE2	↓ VAS pain, WOMAC pain, stiffness, function and total, ↓ Analgesic and NSAIDs use	[21]
Epigallocatechin 3-gallate (from green tea)	400–1000 mg	IL-1β, TNF-α, CCR2, NF-κB, JNK-MAPK, ROS, NO, COX-2, PGE2	↓ VAS pain, WOMAC pain	[22]
Gingerols (from ginger)	250–400 mg ginger (5% gingerols)	iNOS, NF-κB, TRPV1 agonist	↓ VAS pain, WOMAC pain	[23,24]
Glucosamine	500–1500 mg	IL-1, IL-6, TNF-α, IL-1β, TGF-β MMPs,NF-κB, ROS formation, improve of proteoglycans, type II collagen and GAGs expression	↓ VAS pain, WOMAC pain, ↓ Analgesic and NSAIDs use	[17,18,19]
Hyaluronic acid	80–200 mg	CD44 receptors, IL-1β, -6, -9, MMPs, PGE2, TNFα, RHAMM, TLR4, ICAM-1, Nf-kB	↓ VAS pain, WOMAC pain, stiffness, function and total	[25]
Methylsulfonylmethane	500–1500 mg	NF-Κb, IL-1, IL-6, IL-1β, TNF-α ROS, COX	↓ VAS pain, WOMAC pain, stiffness, function and total, ↑ SF-36 quality of life all eight domains including pain, ↑ Global patient and physician assessments of disease status	[26]
Omega-3 (EPA and DHA)	500–4500 mg (EPA + DHA)	NF-κB, COX	↓ Mean WOMAC scores for pain, stiffness, and function, ↓ Analgesic and NSAIDs use, ↓ OA symptoms including morning stiffness, pain in hips and knees	[27,28]
Pycnogenol	100–200 mg	MMPs, COX-1, -2, NF-κB, ROS	↓ VAS pain, WOMAC pain	[29]
Vitamin C	500–2000 mg	MMPs, ROS	↓ VAS pain	[30]
Vitamin D	2000–60000 UI	Bone formation and mineralization, MMPs, osteoclast and osteoblast activity, VEGF	Unclear	[31,32]

CCR2 = C-C chemokine receptor type 2, CD44 = Cluster of differentiation 44, COX = Cyclooxygenase, IL = Interleukin, GAGs = glycosaminoglycans, ICAM-1 = Intercellular adhesion molecule, MMPs = Matrix metalloproteinases, LOX = Lipoxygenase, NF-kB = Nuclear factor-kappa B, NO = Nitric oxide, NSAIDs = Non-steroidal anti-inflammatory drug, OA = Osteoarthritis, PGE2 = Prostaglandin E2, RHAMM = receptor for hyaluronan mediated motility, ROS = Reactive oxygen species, SF-36 = 36-Item short form survey, TGF = Tumor grown factor, TLR4 = Toll like receptor 4, TNF = Tumor necrosis factor, VAS = Visual analogue scale, VEGF = Vascular endothelial growth factor, WOMAC = Western Ontario and McMaster University, ↓ = reduction, ↑ = improvement.
